# Changes in acoustic cardiographic parameters before and after hemodialysis are associated with overall and cardiovascular mortality in hemodialysis patients

**DOI:** 10.1038/s41598-021-81286-5

**Published:** 2021-01-15

**Authors:** Tung-Ling Chung, Yi-Hsueh Liu, Jiun-Chi Huang, Pei-Yu Wu, Szu-Chia Chen, Jer-Ming Chang

**Affiliations:** 1 Graduate Institute of Medicine, College of Medicine, Kaohsiung Medical University, Kaohsiung, Taiwan, ROC; 2 Division of Nephrology, Kaohsiung Veterans General Hospital, Kaohsiung, Taiwan, ROC; 3 Graduate Institute of Clinical Medicine, College of Medicine, Kaohsiung Medical University, Kaohsiung, Taiwan, ROC; 4 Department of Internal Medicine, Kaohsiung Municipal Siaogang Hospital, Kaohsiung Medical University, Kaohsiung, Taiwan, ROC; 5 Division of Cardiology, Department of Internal Medicine, Kaohsiung Medical University Hospital, Kaohsiung Medical University, Kaohsiung, Taiwan, ROC; 6 Division of Nephrology, Department of Internal Medicine, Kaohsiung Medical University Hospital, Kaohsiung Medical University, Kaohsiung, Taiwan, ROC; 7 Faculty of Medicine, College of Medicine, Kaohsiung Medical University, Kaohsiung, Taiwan, ROC

**Keywords:** Biological techniques, Cardiology

## Abstract

Acoustic cardiography can provide simultaneous electrocardiography and acoustic cardiac data to assess the electronic and mechanical heart functions. The aim of this study was to assess whether changes in acoustic cardiographic parameters (ACPs) before and after hemodialysis (HD) are associated with overall and cardiovascular (CV) mortality in HD patients. A total of 162 HD patients was enrolled and ACPs were measured before and after HD, including left ventricular systolic time (LVST), systolic dysfunction index (SDI), third (S3) and fourth (S4) heart sounds, and electromechanical activation time (EMAT). During a follow-up of 2.9 years, 25 deaths occurred with 16 from CV causes. Multivariate analysis showed that high △SDI (per 1; hazard ratio [HR], 2.178; 95% confidence interval [CI], 1.189–3.990), high △EMAT (per 1%; HR, 2.218; 95% CI 1.382–3.559), and low △LVST (per 1 ms; HR, 0.947; 95% CI 0.912–0.984) were independently associated with increased overall mortality. In addition, high △EMAT (per 1%; HR, 2.141; 95% CI 1.117–4.102), and low △LVST (per 1 ms; HR, 0.777; 95% CI 0.637–0.949) were associated with increased CV mortality. In conclusion, the changes in ACPs before and after HD may be a useful clinical marker and stronger prognostic marker of overall and CV mortality than ACPs before HD.

## Introduction

The incidence and prevalence of end-stage renal disease (ESRD) are increasing worldwide. Cardiovascular (CV) disease is common in patients with ESRD, including coronary artery disease, left ventricular (LV) hypertrophy, heart failure and arrhythmias^1,2^, and it is also the major cause of morbidity and mortality in dialysis patients^1^. Cardiac structural and functional abnormalities are also known to increase the risk of CV disease^3,4^, and therefore identifying these risk factors in these patients as early as possible is crucial. Patients with ESRD have a high prevalence of LV dysfunction, which has been associated with an increased risk of sudden death in ESRD patients^5–7^. Cardiac function is most commonly evaluated using echocardiography, however the accuracy of echocardiography is operator-dependent and time consuming.

Acoustic cardiography can provide simultaneous information on both electrocardiography and acoustic cardiac data to assess the electronic and mechanical functions of the heart^8^. Acoustic cardiographic parameters (ACPs) include the measurement of heart sounds and systolic time intervals. Previous studies have demonstrated the use of acoustic cardiography to non-invasively detect increased LV filling pressure and LV systolic dysfunction^9,10^. In addition, it has also been used to detect LV hypertrophy, ischemic heart disease, ventricular fibrillation, heart failure, constrictive pericarditis and sleep apnea^8^.

Our recent study investigated ACPs in hemodialysis (HD) patients before and after HD, and found that the fourth heart sound (S4) and LV systolic time (LVST) were decreased and electromechanical activation time (EMAT) was increased after HD^11^. However, whether changes in ACPs before and after HD (△ACPs) can be used as a prognosis marker remains to be determined. Therefore, the aim of this study was to assess whether △ACPs before and after HD are associated with overall and CV mortality in HD patients.

## Results

The mean age of the included 162 HD patients was 60.4 ± 10.9 years. The clinical characteristics of the survivor and non-survivor groups are shown in Table 1. Compared to the survivors, the non-survivors had a lower serum albumin level, higher △heart rate, lower △PR interval and lower △LVST.

**Table 1:** Comparison of baseline characteristics in patients categorized by survival and mortality.

Characteristics	All patients (n = 162)	Survival (n = 137)	Mortality (n = 25)	*p*
Age	60.4 ± 10.9	60.0 ± 10.8	62.8 ± 11.0	0.240
Male gender (%)	53.1	53.3	52.0	0.906
Smoking (ever *vs.* never)	34.6	35.0	32.0	0.769
Diabetes mellitus (%)	46.3	44.5	56.0	0.290
Hypertension (%)	58.6	56.2	72.0	0.140
Coronary artery disease (%)	9.3	10.2	4.0	0.471
Arteriovenous access type (fistula, %)	90.7	92.0	84.0	0.253
Dialysis vintage (years)	9.1 ± 5.9	9.2 ± 5.9	8.6 ± 6.1	0.616
Systolic blood pressure (mmHg)	157.0 ± 23.3	157.7 ± 23.7	153.5 ± 21.1	0.407
Diastolic blood pressure (mmHg)	81.8 ± 13.5	82.2 ± 13.8	79.2 ± 11.9	0.303
Laboratory parameters				
Albumin (g/dL)	3.9 ± 0.3	3.9 ± 0.3	3.7 ± 0.5	0.018
Triglyceride (mg/dL)	114 (85–181)	121.5 (86.5–182.25)	101 (73–156.5)	0.560
Total cholesterol (mg/dL)	173.1 ± 41.7	175.3 ± 40.7	161.4 ± 45.7	0.126
Hemoglobin (g/dL)	10.5 ± 1.3	10.6 ± 1.3	10.1 ± 1.3	0.057
Total calcium (mg/dL)	9.4 ± 0.9	9.4 ± 0.9	9.4 ± 0.7	0.811
Phosphorous (mg/dL)	4.6 ± 1.0	4.6 ± 1.0	4.5 ± 1.0	0.596
CaXP product (mg^2^/dL^2^)	43.8 ± 9.9	44.0 ± 10.0	42.6 ± 9.2	0.542
Potassium (mEq/L)	4.8 ± 0.6	4.9 ± 0.7	4.7 ± 0.6	0.160
Uric acid (mg/dL)	7.3 ± 1.4	7.3 ± 1.4	7.1 ± 1.5	0.489
Kt/V (Daugirdas)	1.69 ± 0.27	1.68 ± 0.27	1.75 ± 0.29	0.281
Blood flow (ml/min)	279.6 ± 20.1	279.8 ± 19.9	278.4 ± 21.7	0.754
Medications				
ACEI and/or ARB use (%)	26.5	25.5	32.0	0.502
β-blocker use (%)	23.5	22.6	28.0	0.560
Statin use (%)	22.8	22.6	24.0	0.881
Aspirin use (%)	17.3	17.5	16.0	1.000
ACP before hemodialysis and △ACP				
Heart rate (beats/min)	79.2 ± 12.0	79.2 ± 12.0	78.7 ± 12.3	0.844
△Heart rate (beats/min)	− 3.2 ± 0.9	− 4.0 ± 1.0	1.5 ± 2.1	0.033
QRS Duration (ms)	103.5 ± 30.9	103.0 ± 30.4	106.3 ± 33.9	0.630
△QRS Duration (ms)	1.4 ± 2.7	1.4 ± 2.9	1.1 ± 7.9	0.966
QTc (ms)	420.1 ± 37.5	419.1 ± 38.3	425.8 ± 32.9	0.415
△QTc (ms)	− 3.8 ± 4.6	− 5.9 ± 5.0	8.2 ± 10.5	0.269
PR interval (ms)	171.2 ± 34.6	170.3 ± 33.5	176.1 ± 40.2	0.459
△PR interval (ms)	− 9.2 ± 3.7	− 3.6 ± 3.4	− 34.9 ± 11.6	0.017
S3	2.8 ± 1.3	2.8 ± 1.3	2.7 ± 1.1	0.862
△S3	− 0.09 ± 0.11	− 0.10 ± 0.12	− 0.02 ± 0.31	0.788
S4	4.9 ± 2.0	4.9 ± 2.0	4.8 ± 1.8	0.889
△S4	− 1.0 ± 0.2	− 1.0 ± 0.2	− 1.3 ± 0.4	0.468
SDI	3.7 ± 1.6	3.7 ± 1.5	3.8 ± 2.0	0.816
△SDI	0.071 ± 0.123	0.001 ± 0.122	0.460 ± 0.433	0.180
EMAT (ms)	90.9 ± 15.7	91.2 ± 14.8	89.3 ± 20.0	0.580
△EMAT (ms)	4.4 ± 1.2	4.4 ± 1.3	4.4 ± 3.8	0.999
EMAT (%)	11.4 ± 2.8	11.5 ± 2.8	11.1 ± 2.9	0.485
△EMAT (%)	0.08 ± 0.23	− 0.05 ± 0.26	0.85 ± 0.59	0.167
LVST (ms)	331.8 ± 37.5	331.8 ± 37.7	331.7 ± 37.2	0.988
△LVST (ms)	− 11.9 ± 3.9	− 7.8 ± 3.6	− 34.9 ± 15.0	0.012
LVST (%)	42.4 ± 4.6	42.4 ± 4.6	42.4 ± 5.1	0.975
△LVST (%)	− 2.9 ± 0.4	− 3.0 ± 0.4	− 2.0 ± 1.2	0.400

Abbreviation: CaXP product, calcium × phosphorus product; ACEI, angiotensin converting enzyme inhibitor; ARB, angiotensin II receptor blocker; ACP, acoustic cardiography parameters; SDI, Systolic dysfunction index; EMAT, Electromechanical activation time; LVST, LV systolic time.

### Determinants of △ACPs

Table 2 shows the determinants of △ACPs using multivariable linear regression analysis. In the multivariable analysis after adjusting for age, sex, diabetes mellitus, hypertension, coronary artery disease, arteriovenous access type, dialysis vintage, systolic and diastolic blood pressures, albumin, log triglyceride, total cholesterol, hemoglobin, total calcium, phosphorous, calcium × phosphorus (CaXP) product, potassium, uric acid, Kt/V, blood flow, and medications including angiotensin converting enzyme inhibitors (ACEIs) and/or angiotensin II receptor blockers (ARBs), β-blocker, statin and aspirin, diabetes, high triglyceride, high hemoglobin, and patients withoutβ-blocker use were significantly associated with high △heart rate. Diabetes was significantly associated with high △QRS Duration. Hypertension, high potassium, and patients without ACEI and/or ARB use were related with low △PR interval. High systolic blood pressure was correlated with low △S3, and low blood flow was associated with △S4. Diabetes and low systolic blood pressure were associated with high △SDI. Low systolic blood pressure, high diastolic blood pressure, high total calcium, high phosphorous, low CaXP product were associated with high △EMAT. Low systolic blood pressure, high diastolic blood pressure, high triglyceride, high total calcium, high phosphorous, low CaXP product were associated with high △EMAT%. Lastly, low systolic blood pressure, high diastolic blood pressure, high triglyceride, high total calcium, and low blood flow were associated with low △LVST. There were no significant variables associated with △QTc and △LVST%.

**Table 2:** Relation of using multivariable linear regression analysis.

ACP before hemodialysis and △ACP	Multivariable	
	Unstandardized coefficient β (95% CI)	*p*
△Heart rate (beats/min)		
Diabetes mellitus	5.886 (0.839, 10.934)	0.023
Triglyceride (log per 1 mg/dL)	12.431 (2.033, 22.828)	0.020
Hemoglobin (per 1 g/dL)	2.165 (0.381, 3.948)	0.018
β-blocker use	− 6.241 (− 12.069, − 0.412)	0.036
△QRS Duration (ms)		
Diabetes mellitus	19.241 (2.648, 35.833)	0.024
△PR interval (ms)		
Hypertension	− 22.306 (− 40.933, − 3.680)	0.020
Potassium (per mEq/L)	− 12.568 (− 23.980, − 1.157)	0.031
ACEI and/or ARB use	27.216 (2.739, 51.693)	0.030
△S3		
Systolic blood pressure (per 1 mmHg)	− 0.020 (− 0.041, 0)	0.046
△S4		
Blood flow (per 1 ml/min)	0.022 (0.005, 0.038)	0.011
△SDI		
Diabetes mellitus	1.028 (0.287, 1.770)	0.007
Systolic blood pressure (per 1 mmHg)	− 0.027 (− 0.050, − 0.003)	0.025
△EMAT (ms)		
Systolic blood pressure (per 1 mmHg)	− 0.284 (− 0.495, − 0.073)	0.009
Diastolic blood pressure (per 1 mmHg)	0.661 (0.256, 1.067)	0.002
Total calcium (per 1 mg/dL)	10.666 (1.109, 20.223)	0.029
Phosphorous (per 1 mg/dL)	18.008 (1.027, 34.988)	0.038
CaXP product (per 1 mg^2^/dL^2^)	− 1.944 (-3.863, − 0.025)	0.047
△EMAT (%)		
Systolic blood pressure (per 1 mmHg)	− 0.056 (− 0.095, − 0.017)	0.006
Diastolic blood pressure (per 1 mmHg)	0.112 (13,167, 0.187)	0.004
Triglyceride (log per 1 mg/dL)	2.793 (0.222, 5.363)	0.034
Total calcium (per 1 mg/dL)	2.123 (0.355, 3.891)	0.019
Phosphorous (per 1 mg/dL)	3.635 (0.494, 6.776)	0.024
CaXP product (per 1 mg^2^/dL^2^)	− 0.399 (− 0.754, − 0.044)	0.028
△LVST (ms)		
Systolic blood pressure (per 1 mmHg)	0.562 (0.040, 1.084)	0.035
Diastolic blood pressure (per 1 mmHg)	− 1.119 (− 2.121, − 0.117)	0.029
Triglyceride (log per 1 mg/dL)	− 53.344 (− 87.722, − 18.966)	0.003
Total calcium (per 1 mg/dL)	− 24.414 (− 48.055, − 0.772)	0.043
Blood flow (per 1 ml/min)	0.341 (0.022, 0.660)	0.036

Values expressed as unstandardized coefficient β and 95% confidence interval (CI). Abbreviations are the same as in Table 1.

Covariates in the multivariable model included age, sex, diabetes mellitus, hypertension, coronary artery disease, arteriovenous access type, dialysis vintage, systolic and diastolic blood pressures, albumin, log triglyceride, total cholesterol, hemoglobin, total calcium, phosphorous, CaXP product, potassium, uric acid, Kt/V, blood flow, and medications including ACEIs and/or ARBs, β-blocker, statin and aspirin.

### Risk of overall mortality

The median follow-up period was 2.9 (range 2.4–3.4) years for all patients, during which 25 patients died (15.4%), including CV death (n = 16), malignancy (n = 3), infectious disease (n = 3), and others (n = 3). Table 3 shows the relationships between ACPs before HD and △ACPs to overall mortality. In the multivariable analysis after adjustment, high △heart rate (per 1 beat/min; hazard ratio [HR], 1.148; 95% confidence interval [CI], 1.053–1.253; *p* = 0.002), low △PR interval (per 1 ms; HR, 0.930; 95% CI 0.888–0.975; *p* = 0.002), high △SDI (per 1; HR, 2.178; 95% CI 1.189–3.990; *p* = 0.012), high EMAT (per 1%; HR, 1.508; 95% CI 1.056–2.154; *p* = 0.024), high △EMAT (per 1%; HR, 2.218; 95% CI 1.382–3.559; *p* = 0.001), low LVST (per 1 ms; HR, 0.9679; 95% CI 0.946–0.994; *p* = 0.014), and low △LVST (per 1 ms; HR, 0.947; 95% CI 0.912–0.984; *p* = 0.005) were independently associated with increased overall mortality.

**Table 3:** Relation of acoustic cardiography parameters before hemodialysis and △acoustic cardiography parameters to overall mortality.

ACP before hemodialysis and △ACP	Multivariable	
	Hazard ratio (95% CI)	*p*
Heart rate (per 1 beats/min)	1.041 (0.970–1.118)	0.265
△Heart rate (per 1 beats/min)	1.148 (1.053–1.253)	0.002
QRS Duration (per 1 ms)	1.005 (0.973–1.038)	0.763
△QRS Duration (per 1 ms)	1.009 (0.980–1.039)	0.556
QTc (per 1 ms)	1.012 (0.997–1.048)	0.079
△QTc (per 1 ms)	1.016 (0.997–1.035)	0.090
PR interval (per 1 ms)	1.037 (0.965–1.116)	0.323
△PR interval (per 1 ms)	0.930 (0.888–0.975)	0.002
S3 (per 1)	0.819 (0.393–1.704)	0.593
△S3 (per 1)	1.258 (0.651–2.432)	0.495
S4 (per 1)	0.900 (0.453–1.790)	0.765
△S4 (per 1)	0.799 (0.384–1.662)	0.548
SDI (per 1)	1.635 (0.865–3.090)	0.130
△SDI (per 1)	2.178 (1.189–3.990)	0.012
EMAT (per 1 ms)	1.044 (0.981–1.111)	0.176
△EMAT (per 1 ms)	1.075 (0.987–1.171)	0.095
EMAT (per 1%)	1.508 (1.056–2.154)	0.024
△EMAT (per 1%)	2.218 (1.382–3.559)	0.001
LVST (per 1 ms)	0.969 (0.946–0.994)	0.014
△LVST (per 1 ms)	0.947 (0.912–0.984)	0.005
LVST (per 1%)	1.037 (0.857–1.255)	0.712
△LVST (per 1%)	1.093 (0.890–1.342)	0.394

Values expressed as hazard ratio and 95% confidence interval (CI). Abbreviations are the same as in Table 1.

Covariates in the multivariable model included age, sex, diabetes mellitus, hypertension, coronary artery disease, arteriovenous access type, dialysis vintage, systolic and diastolic blood pressures, albumin, log triglyceride, total cholesterol, hemoglobin, total calcium, phosphorous, CaXP product, potassium, uric acid, Kt/V, blood flow, and medications including ACEIs and/or ARBs, β-blocker, statin and aspirin.

**Figure 1:**
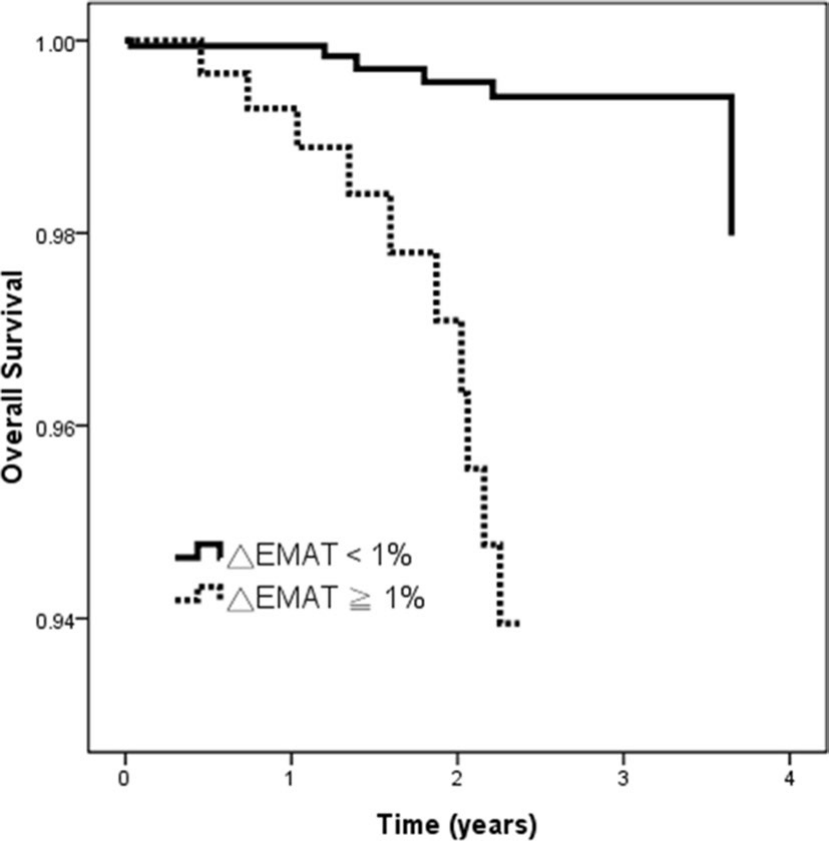
Adjusted overall survival curves in patients according to 75th percentile of △EMAT (1%). Patients with △EMAT ≧ 1% had a worse overall survival than those with △EMAT < 1% (*p* = 0.015).

The adjusted Cox regression survival curves [adjusted for age, sex, diabetes mellitus, hypertension, coronary artery disease, arteriovenous access type, dialysis vintage, systolic and diastolic blood pressures, albumin, log triglyceride, total cholesterol, hemoglobin, total calcium, phosphorous, CaXP product, potassium, uric acid, Kt/V, blood flow, and medications including ACEIs and/or ARBs, β-blocker, statin and aspirin] for overall survival in the patients according to the 75th percentile of △EMAT (1%) are shown in Fig. 1. Patients with △EMAT ≥ 1% had a worse overall survival than those with △EMAT < 1% (HR, 9.554; 95% CI 1.560–58.518; *p* = 0.015).

**Figure 2:**
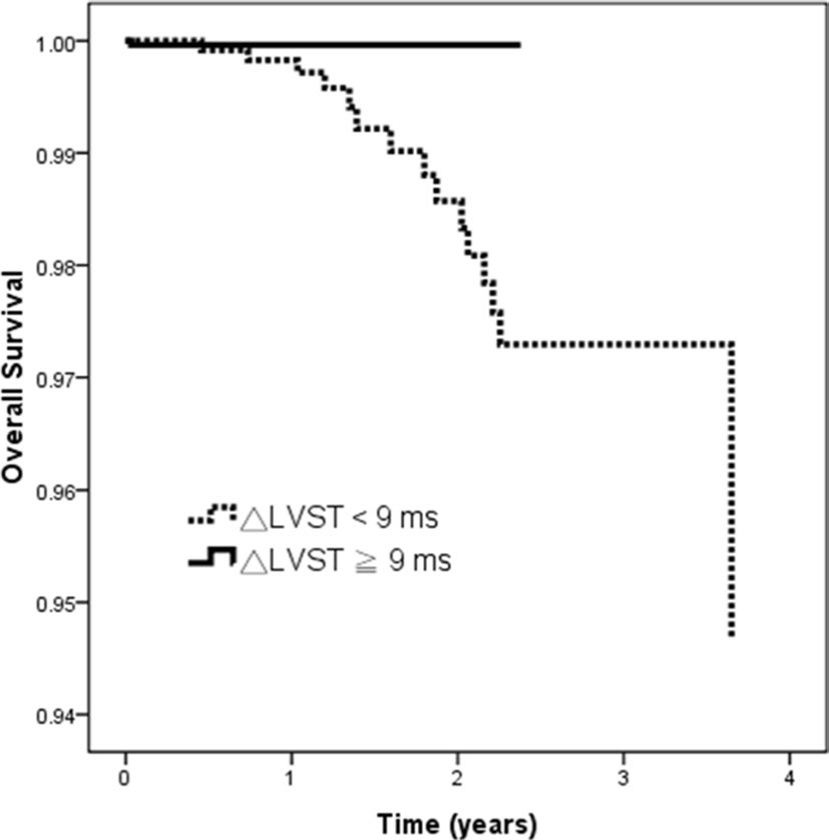
Adjusted overall survival curves in patients according to 75th percentile of △LVST (9 ms). Patients with △LVST < 9 ms had a worse overall survival than those with △LVST ≧ 9 ms (*p* = 0.015).

The adjusted Cox regression survival curves for overall survival in the patients according to the 75th percentile of △LVST (9 ms) are shown in Fig. 2. The patients with △LVST < 9 ms had a worse overall survival than those with △LVST ≥ 9 ms (HR, 0.018; 95% CI 0.001–0.455; *p* = 0.015).

### Risk of CV mortality

There were 16 documented CV deaths during follow-up, including heart failure (n = 7), myocardial infarction (n = 3), ventricular fibrillation (n = 4) and hemorrhagic stroke (n = 2). Table 4 lists the predictors for CV mortality after adjusting for demographic, clinical, and biochemical factors, and ACPs before HD and △ACPs. Multivariable analysis revealed that low △PR interval (per 1 ms; HR, 0.910; 95% CI 0.846–0.980; *p* = 0.012), high △EMAT (per 1%; HR, 2.141; 95% CI 1.117–4.102; *p* = 0.022), low LVST (per 1 ms; HR, 0.837; 95% CI 0.730–0.960; *p* = 0.011), and low △LVST (per 1 ms; HR, 0.777; 95% CI 0.637–0.949; *p* = 0.013) were significantly associated with increased CV mortality.

**Table 4:** Relation of acoustic cardiography parameters before hemodialysis and △acoustic cardiography parameters to cardiovascular mortality.

ACP before hemodialysis and △ACP	Multivariable	
	Hazard ratio (95% CI)	*p*
Heart rate (per 1 beats/min)	0.996 (0.770–1.289)	0.978
△Heart rate (per 1 beats/min)	1.526 (0.856–2.720)	0.152
QRS Duration (per 1 ms)	0.989 (0.926–1.055)	0.734
△QRS Duration (per 1 ms)	1.001 (0.944–1.062)	0.962
QTc (per 1 ms)	1.017 (0.980–1.055)	0.372
△QTc (per 1 ms)	1.015 (0.981–1.050)	0.394
PR interval (per 1 ms)	1.938 (0.862–1.020)	0.135
△PR interval (per 1 ms)	0.910 (0.846–0.980)	0.012
S3 (per 1)	0.663 (0.206–2.134)	0.491
△S3 (per 1)	2.273 (0.619–8.339)	0.216
S4 (per 1)	0.300 (0.036–2.481)	0.264
△S4 (per 1)	0.507 (0.051, 5.049)	0.582
SDI (per 1)	2.498 (0.590–10.565)	0.213
△SDI (per 1)	3.273 (0.699–15.328)	0.132
EMAT (per 1 ms)	1.059 (0.963–1.166)	0..237
△EMAT (per 1 ms)	1.066 (0.937–1.213)	0.334
EMAT (per 1%)	1.539 (0.954–2.482)	0.077
△EMAT (per 1%)	2.141 (1.117–4.102)	0.022
LVST (per 1 ms)	0.837 (0.730–0.960)	0.011
△LVST (per 1 ms)	0.777 (0.637–0.949)	0.013
LVST (per 1%)	0.893 (0.668–1.194)	0.445
△LVST (per 1%)	1.135 (0.830–1.554)	0.428

Values expressed as hazard ratio and 95% confidence interval (CI). Abbreviations are the same as in Table 1.

Covariates in the multivariable model included age, sex, diabetes mellitus, hypertension, coronary artery disease, arteriovenous access type, dialysis vintage, systolic and diastolic blood pressures, albumin, log triglyceride, total cholesterol, hemoglobin, total calcium, phosphorous, CaXP product, potassium, uric acid, Kt/V, blood flow, and medications including ACEIs and/or ARBs, β-blocker, statin and aspirin.

## Discussion

To the best of our knowledge, this is the first study to report associations between changes in ACP before and after HD with overall and CV mortality. Our results showed that high △heart rate, low △PR interval, high △SDI, high EMAT, high △EMAT, low △LVST, and low △LVST were significantly associated with increased overall mortality. In addition, low △PR interval, high △EMAT, low LVST and low △LVST were significantly associated with increased CV mortality.

The first important finding of this study is that high △EMAT before and after HD was associated with increased overall and CV mortality. EMAT is defined as the amount of time required for the left ventricle to generate sufficient force to close the mitral valve, and therefore reflects the velocity of force generation during systole^12^. Previous studies have shown a strong association between EMAT and impaired LV contractility, and that EMAT increases steadily with age in patients with heart failure^13,14^. EMAT has also been shown to be a useful prognostic marker of CV outcomes. In a study of hospitalized patients with congestive heart failure, Zhang et al. found that elevated EMAT measured at admission was an independent risk factor for major adverse CV events^15^. In patients with acute heart failure syndrome, Chao et al. reported that EMAT could predict CV outcomes independently of LV ejection fraction, LV diastolic function, and serum N-terminal pro-brain natriuretic peptide^16^. In addition, Sung et al. reported that a high %EMAT or S3 strength may indicate an increased risk of de-compensation and mortality in patients with heart failure, and that intensifying diuretic and/or vasodilator therapy may alleviate potential adverse events^17^. But why did the EMAT increase after HD in our study? The potential mechanism may be insufficient coronary perfusion and cause resulting heart ischemia and impaired heart systolic function. Furthermore, there may be a stenosis lesion on coronary artery, so the coronary perfusion is susceptible to volume depletion (decrease in effective circulating volume) by ultrafiltration. In patients with elevated EMAT after HD, this may contribute to higher cardiovascular risk. In the present study, △EMAT was a stronger predictor of adverse outcomes than EMAT before HD. Therefore, dynamic changes in EMAT before and after HD may reflect impaired LV contractility more accurately than resting APCs before HD.

The second important finding of this study is that low △LVST before and after HD was associated with increased overall and CV mortality. In 1970, Garrard et al. reported a significant correlation between LVST and angiographically determined LV ejection fraction and left ventricular end-diastolic volume^18^. Patients with LV systolic dysfunction often have a shorter LVST as more time is required to generate sufficient force to open the aortic valve and keep it open in situations with impaired ventricular contraction^19^. Zuber et al. also showed that patients with an LV ejection fraction < 50% had a higher prevalence of S3, lower mean LVST, and higher mean EMAT and EMAT/LVST. The authors concluded that these parameters can improve detection of LV systolic dysfunction^20^. In addition, in patients with intermediate brain natriuretic peptide levels (100 to 500 pg/mL), Shapiro et al. showed that EMAT/LVST could detect LV dysfunction with a high specificity (95%) and moderate sensitivity (55%) and improve the detection of LV dysfunction^21^. In the present study, we found that low △LVST was associated with increased overall and CV mortality, and that this was a stronger predictor of adverse outcomes than LVST before HD.

Another important finding of this study is that high △SDI before and after HD was correlated with increased overall mortality. In patients with heart failure, previous studies have shown that SDI can discriminate patients with severe LV systolic dysfunction (ejection fraction [EF] ≤ 35%) from those with moderate LV systolic dysfunction (35% < EF < 50%) with an SDI > 5^22^. Dillier et al. reported that both SDI (≥ 5) and S3 (4) score were strong independent predictors of mortality and could identify high-risk patients requiring more aggressive monitoring and therapy^23^. In the present study, we found that high △SDI was associated with higher overall mortality, and that it was a stronger predictor of adverse outcomes than SDI before HD.

In this study, the patients with an increased heart rate after HD had higher overall mortality. The reason for an increase in heart rate after HD is not fully understood, but it may be related to progressive volume depletion by ultrafiltration, sympatho-activation and changes in electrolyte levels^24,25^. The autonomic system controls heart rate and rhythm via a balance between parasympathetic and sympathetic systems^26^. A previous study reported that HD patients are characterized by vagal withdrawal and sympathetic overactivity, and that this was linked to a poor CV prognosis^27^. In addition, Barnas et al. investigated hemodynamic patterns during dialysis hypotension, and demonstrated that tachycardia induced by ultrafiltration is part of the spectrum of normal CV autonomic activation. The authors concluded that tachycardia develops by increasing baroreflex-mediated sympathetic activity^24^. Rogovoy et al. investigated sudden cardiac death in ESRD patients using continuous electrocardiography monitoring, and found that sudden increases in heart rate during or after dialysis preceded non-sustained ventricular tachycardia events^26^. In addition, Severi et al. reported a significant increase (11%) in heart rate following changes in calcium, potassium and pH, without significant changes in autonomic activity indices^25^.

Studies on the association between PR interval and mortality have reported inconsistent results. Magnani et al. conducted a prospective cohort study with 2722 older participants (age 70–79 years), and multivariable-adjusted analyses showed that every SD increase (29 ms) in baseline PR interval was associated with 13% increases in the 10-year risk of both incident atrial fibrillation and heart failure. However, they did not find an association between PR interval and all-cause mortality^28^. Skampardoni et al. published a review of patients with chronic kidney disease or dialysis, and showed varying results of the association between PR interval prolongation and CV outcomes and overall mortality^29^. Moreover, Flueckiger et al. reported an association between prolonged PR interval and increased mortality^30^, while Kestenbaum et al. reported that a longer PR interval could not independently predict incident CV events or mortality^31^. In addition, Silva et al. reported that prolonged PR interval could predict bradyarrhythmias^32^. Regarding the difference between PR intervals before and after HD, Badarau et al. reported that an increased PR interval after HD was associated with lower rates of CV events and mortality^33^. During dialysis, changes in electrolyte levels, fluid shift and autonomic response may influence the PR interval^29^. In the present study, the patients who had low △PR interval before and after HD were associated with increased overall and CV mortality. Therefore, further studies are needed to investigate the significance and prognostic value of decreased PR interval after dialysis.

There are several limitations to this study. First, because relatively few patients died, the statistical power of the study was reduced. Further studies with a larger sample size should be performed to verify our findings. In addition, the quality of acoustic cardiography data is influenced by exogenous and endogenous noises. Finally, the ACPs were measured only before and after HD, and repeated measurements may be necessary.

In conclusion, we demonstrated that high △SDI, high △EMAT and low △LVST were independently associated with increased overall mortality. In addition, high △EMAT and low △LVST were significantly associated with increased CV mortality. Changes in ACP before and after HD (△ACP) may be a useful clinical marker and stronger prognostic marker of overall and CV mortality than ACPs before HD. Acoustic cardiography is a non-invasive cost-effective technique to screen dialysis patients who are at high risk of adverse clinical outcomes.

## Materials and methods

### Ethics statement

Written informed consent was obtained from each participant in accordance with institutional requirements and the principles of the Declaration of Helsinki. Moreover, the current study was approved by the Institutional Review Board of Kaohsiung Medical University Hospital (KMUHIRB-E(I)-20160071).

### Study patients and design

This study was conducted at the dialysis unit of a regional hospital in southern Taiwan from October 2016 to April 2018, and enrolled 162 patients (86 [53.1%] males, 76 females [46.9%]) receiving HD treatment three times a week. Each HD session lasted for 3.5 to 4 h. The dialyzer blood flow rates ranged from 250 to 300 mL/min with a dialysate flow rate of 500 mL/min. The study protocol was approved by the Institutional Review Board of Kaohsiung Medical University Hospital, and informed consent was obtained from all of the enrolled patients.

### Demographic, medical, and laboratory data collection

Age, sex, smoking status (ever *vs.* never) and comorbid conditions of the patients were obtained from medical records and interviews. After overnight fasting, blood samples were obtained and biomarkers including triglycerides, hemoglobin, albumin, fasting glucose, uric acid, and total cholesterol, calcium, phosphorus, and potassium were measured. The efficiency of dialysis was determined by Kt/V according to the Daugirdas method^34^. All blood samples were obtained within 1 month of enrollment into the study.

### Acoustic cardiography

Acoustic cardiographic examinations were performed in the supine position 30 min before and 30 min after a mid-weekly HD session. Acoustic cardiographic data including heart sounds and related systolic time intervals were analyzed using an AUDICOR system (Inovise Medical). The following ACPs were measured^8,12,22^:

1. EMAT and %EMAT: the time from Q wave onset to the mitral component of the first heart sound (S1). EMAT was defined as the time required for the left ventricle to generate sufficient force to close the mitral valve.
2. LVST: the time from S1 to the second heart sound (S2).
3. The third heart sound (S3): the probability that S3 existed. A single value between 0 and 10 was reported, and the presence of S3 was defined as a value ≥ 5.
4. S4: as with S3, a single value between 0 and 10 was reported, and the presence of S4 was defined as a value ≥ 5.
5. Systolic dysfunction index (SDI): SDI was derived from the nonlinear transformation of [(S3 score ÷ 10) × QRS duration × QR interval × %EMAT] and mapped onto a scale of 0 to 10.

### Definition of overall and CV mortality

Cases of CV and overall mortality were verified from medical records by two cardiologists, with disagreements being resolved by a third cardiologist. Patients were followed until death or September 2020. Patients who did not die were censored at the end of follow-up.

### Statistical analysis

Data are expressed as percentages, mean ± standard deviation for ACPs, mean ± standard error of mean for △ACP, or median (25th–75th percentile) for triglycerides. The patients were divided into two groups as survivors and non-survivors. Between-group differences in categorical variables were assessed using the chi-square test. Approximately normally distributed continuous variables were analyzed using the independent t-test, and continuous variables with skewed distribution were analyzed using the Mann–Whitney U test. △ACP was defined as the difference between the ACP measurement before and after HD. Multivariable linear regression analysis was used to identify the determinants for △ACP. Multivariable Cox proportional hazard analysis was used to identify associations between an ACP before HD and △ACP and overall and CV mortality. Survival curves for overall and CV mortality were derived using Cox-regression analysis. A *p* value < 0.05 was considered to be statistically significant. Statistical analyses were performed using SPSS 19.0 for Windows (SPSS Inc. Chicago, USA).
